# Editorial for the Special Issue “Molecular Mechanisms and Treatment of Ischemia–Reperfusion Injury”

**DOI:** 10.3390/cimb48080799

**Published:** 2026-08-06

**Authors:** Jun Kobayashi

**Affiliations:** Department of Clinical Dietetics and Human Nutrition, Faculty of Pharmaceutical Science, Josai University, 1-1 Keyakidai, Sakado 350-0295, Japan; junkoba@josai.ac.jp

Ischemia–reperfusion injury (IRI) remains one of the most challenging obstacles in modern medicine, influencing outcomes across cardiovascular disease, organ transplantation, stroke, peripheral ischemia, and acute surgical conditions. Although restoration of blood flow is essential for tissue survival, reperfusion paradoxically initiates a cascade of oxidative stress, inflammation, mitochondrial dysfunction, metabolic reprogramming, and multiple forms of regulated cell death. Consequently, the development of effective therapies requires a comprehensive understanding of the complex molecular networks underlying IRI.

The studies collected in this Special Issue provide an updated perspective on the evolving landscape of IRI research. Together, they demonstrate how advances in immunology, metabolism, non-coding RNA biology, ferroptosis, autophagy, gene therapy, and antioxidant interventions are reshaping our understanding of tissue injury and repair.

One of the central themes emerging from this collection is the growing appreciation of inflammation as a dynamic regulator rather than simply a secondary consequence of reperfusion injury. Robertson et al. provided valuable clinical evidence from liver transplantation demonstrating rapid recruitment of recipient-derived inflammatory monocytes into the transplanted liver immediately following reperfusion [1,2]. Importantly, elevated cytokines associated with innate immune activation correlated with graft-specific complications, highlighting inflammatory monocytes as promising therapeutic targets. Unlike many experimental studies, this work directly investigates immune responses in human transplantation, strengthening the translational relevance of innate immune modulation.

Inflammatory signaling also plays a pivotal role in cerebral IRI. Ayaz et al. demonstrated that Skimmianine (furoquinoline alkaloid) significantly attenuated oxidative stress while suppressing microglial activation and inflammatory mediators including IBA-1 (ionized calcium-binding adapter molecule 1), IL-6 (Interleukin-6), and NF-κB following cerebral ischemia [3]. These findings support the concept that neuroprotection requires the simultaneous control of oxidative damage and neuroinflammation rather than targeting either process alone [4].

Oxidative stress remains a hallmark of IRI irrespective of the affected organ. Several contributions have explored antioxidant strategies using naturally occurring compounds or clinically available agents. Seğmen et al. showed that vitamin B complex, particularly when combined with alpha-lipoic acid, improved antioxidant capacity and reduced biochemical evidence of hepatic injury in a rat liver IRI model [5,6]. Likewise, Karateke et al. demonstrated that combined administration of mannitol and vitamin D provided superior protection against ovarian torsion-induced IRI compared with either treatment alone [7,8], reducing oxidative stress while preserving follicular integrity and cellular proliferation [7]. Collectively, these findings support the concept that combination therapies targeting multiple oxidative pathways may provide greater efficacy than monotherapy.

Natural products also continue to emerge as promising therapeutic candidates. Liu et al. identified an active extract from *Allium chinense* that was capable of attenuating myocardial IRI by inhibiting platelet activation [9]. Through integrated transcriptomic analysis, molecular docking, and experimental validation, the investigators identified PDK1 and PKM2, key regulators of aerobic glycolysis, as potential molecular targets. The discovery that sarsasapogenin and hecogenin interact with these metabolic regulators illustrates the increasing convergence between platelet biology, cellular metabolism, and ischemic injury.

Beyond oxidative stress and inflammation, regulated cell death has become an increasingly important therapeutic focus. While apoptosis has long been recognized in IRI, several papers highlight the importance of alternative cell death pathways.

Yu et al. identified a novel miR-451a/KLF1/ACSL4 (microRNA 451a/Krüppel-like factor 1/acyl-CoA synthetase long-chain family member 4) signaling axis regulating ferroptosis during renal IRI [10,11]. Increased miR-451a expression suppressed KLF1, resulting in enhanced ACSL4 expression and accelerated ferroptotic injury. These findings extend growing evidence that ferroptosis contributes substantially to delayed graft function following kidney transplantation and suggest that modulation of ferroptotic signaling could improve transplant outcomes.

Autophagy represents another major focus throughout this Special Issue. Park et al. demonstrated that inhibition of macrophage migration inhibitory factor (MIF) reduced autophagic activity in astrocytes exposed to hypoxic injury, suggesting that MIF contributes to adaptive autophagic responses during ischemia [12]. In contrast, Zakharova et al. showed that intranasal insulin reduced excessive neuronal autophagy after global cerebral ischemia through the activation of Akt/mTOR signaling and inhibition of AMPK, thereby improving neuronal survival [13]. Together, these observations underscore the dual nature of autophagy in IRI: physiological autophagy may initially promote cellular adaptation, whereas excessive activation ultimately contributes to neuronal death [14]. Therapeutic interventions therefore require careful temporal regulation rather than complete inhibition.

The regulatory complexity of autophagy is further explored in the comprehensive review by Zakharova et al. [15]. The authors summarize how long non-coding RNAs, microRNAs, and circular RNAs interact through competitive endogenous RNA networks to regulate autophagy-related genes during cerebral IRI [16]. Rather than acting independently, these RNA species form interconnected signaling axes that fine-tune autophagic responses. Their review highlights ncRNA-based therapeutics as an emerging frontier for precision medicine.

Similarly, Xu et al. reviewed the expanding role of non-coding RNAs in regulating pyroptosis during cerebral ischemia–reperfusion [17]. Pyroptosis has emerged as an inflammatory form of programmed cell death distinct from apoptosis and ferroptosis [18]. The authors demonstrate how ncRNAs regulate inflammasome activation and pyroptotic signaling across neurons, astrocytes, microglia, and endothelial cells. Together with the review by Zakharova et al., these articles emphasize that RNA-based regulation represents one of the fastest-growing research areas in IRI biology.

While molecular mechanisms dominate much of the current literature, regenerative approaches are equally important for restoring tissue function after ischemia. Samatoshenkov et al. investigated combinatorial gene delivery using VEGF, angiopoietin, and GDNF in chronic limb ischemia [19,20]. Their results demonstrated enhanced angiogenesis, improved reinnervation, and accelerated skeletal muscle regeneration, particularly when gene delivery was performed using umbilical cord blood mononuclear cells. These findings illustrate how regenerative medicine may complement traditional cytoprotective approaches by actively promoting vascular and neural repair after ischemic injury.

Kidney transplantation continues to represent one of the clinical settings in which IRI has the greatest impact. Huang et al. provided a timely review summarizing current knowledge regarding mitochondrial dysfunction, oxidative stress, programmed cell death, and emerging therapeutic interventions including machine perfusion, ischemic conditioning, nanoparticles, peptide therapeutics, and microRNA-based approaches [21,22]. Their synthesis reflects the growing shift toward multimodal strategies that target multiple mechanisms simultaneously rather than relying on single-agent interventions.

Taken together, this Special Issue highlights several overarching themes. IRI is increasingly recognized as an integrated pathological process involving immune activation, metabolic remodeling, mitochondrial dysfunction, and multiple forms of regulated cell death. Emerging regulatory networks, particularly those involving non-coding RNAs and metabolic signaling, are expanding opportunities for mechanism-based therapeutic intervention. Across different organ systems, the studies consistently support multimodal therapeutic strategies and emphasize the importance of translating mechanistic insights into clinically relevant applications.

Collectively, the studies presented in this Special Issue highlight the rapid evolution of IRI research from descriptive studies of tissue injury toward mechanism-driven therapeutic development. Advances in systems biology, single-cell and spatial omics, and precision molecular profiling are expected to further clarify the complex cellular interactions underlying ischemia–reperfusion injury. Integrating these emerging technologies with rigorous translational and clinical investigation will be essential for identifying biomarkers, refining patient stratification, and developing personalized therapeutic strategies. Such efforts will ultimately help bridge the gap between experimental discoveries and effective clinical interventions for ischemic diseases across multiple organ systems.
